# Kutanes Angiosarkom mit klinischem Bild eines Quincke-Ödems

**DOI:** 10.1007/s00105-020-04748-3

**Published:** 2021-01-13

**Authors:** M. Albrecht, E. Hadaschik, L. Zimmer, E. Livingstone, R. Hamacher, S. Bauer, D. Schadendorf, S. Ugurel

**Affiliations:** 1 Klinik und Poliklinik für Dermatologie, Venerologie und Allergologie, Universitätsklinikum Essen, Universität Duisburg-Essen, Essen, Deutschland; 2 Sarkomzentrum, Westdeutsches Tumorzentrum Essen, Universitätsklinikum Essen, Universität Duisburg-Essen, Essen, Deutschland

**Keywords:** Eingeschränkte Operabilität, Paclitaxel, Liposomales Doxorubicin, Pazopanib, Immuncheckpointinhibitoren, Limited operability, Paclitaxel, Liposomal doxorubicin, Pazopanib, Immune checkpoint inhibitors

## Abstract

Es wird über den Fall eines 75-jährigen Patienten mit einer Gesichtsschwellung v. a. periorbital berichtet, der unter der Verdachtsdiagnose eines Quincke-Ödems stationär aufgenommen wurde. Probebiopsien ergaben das Vorliegen eines kutanen Angiosarkoms. Bei nicht resezierbarem Befund und schwieriger Bestrahlungssituation wurde zunächst eine Chemotherapie eingeleitet. Im Verlauf erfolgte bei Befundprogress die Therapieumstellung auf Zweit- und Drittlinientherapie. Der geschilderte Fall verdeutlicht die Komplexität bei der Diagnostik und Therapie bei Patienten mit kutanem Angiosarkom.

## Abstract (en)

We report a case of a 75-year-old man with facial edema that also affected the periorbital area who was admitted to the hospital with the suspected diagnosis of Quincke’s edema. The diagnosis of cutaneous angiosarcoma was made by microscopic examination and immunohistochemical staining. Chemotherapy was initially initiated because the angiosarcoma was unresectable and the radiation situation was difficult. Therapy has to be switched to second and third line therapy due to disease progression. The case illustrates the complexity of diagnosis and therapy in patients with cutaneous angiosarcoma.

## Falldarstellung

### Anamnese

Ein 75-jähriger Patient stellte sich 2017 erstmalig mit einer seit ca. 4 Wochen aufgefallenen Gesichtsschwellung in unserer Klinik vor. Unter der Verdachtsdiagnose eines Quincke-Ödems war bereits eine ambulante Therapie mit Steroiden und Antihistaminika per os eingeleitet worden – zum Zeitpunkt der Vorstellung ohne nachhaltigen Therapieerfolg.

### Klinischer Befund

**Figure 1:**
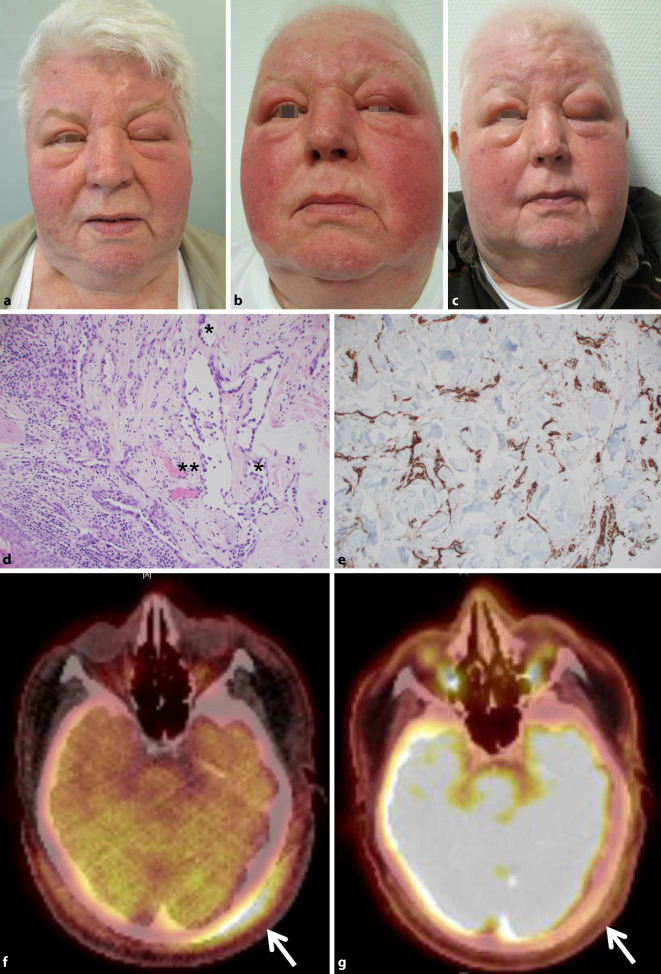

Der Patient präsentierte sich in gutem Allgemeinzustand mit einem ausgeprägten Ödem und Erythem der gesamten Gesichtshaut unter Betonung der oberen Gesichtshälfte, insbesondere der Ober- und Unterlider, links etwas ausgeprägter als rechts (Abb. 1a). Die Hautveränderungen setzten sich bis auf das frontale und hoch parietale Kapillitium fort.

### Verdachtsdiagnose

Unter der Verdachtsdiagnose eines ambulant therapierefraktären Quincke-Ödems wurde der Patient stationär aufgenommen, und es wurde eine intensivierte intravenöse Therapie mit Antihistaminika (2 mg Clemastin 2‑mal täglich) und Prednisolon (100 mg 1‑mal täglich) eingeleitet. Nach 2 Tagen eines unveränderten Hautbefundes wurde eine Probebiopsie aus dem prominentesten Schwellungsareal an der Stirn entnommen.

### Histologie

Der histologische Befund zeigte über das gesamte Korium verteilt zahlreiche schlitzförmige Gefäßspalten mit prominentem Endothel und Zellatypien (Abb. 1d). In der immunhistologischen Untersuchung waren die atypischen Zellen positiv für CD31 und D2–40 sowie negativ für HHV‑8 (Abb. 1e). Weiterhin wiesen sie eine sehr hohe proliferative Aktivität auf (Ki-67 >60 %). In der Zusammenschau der Befunde wurde somit ein kutanes Angiosarkom diagnostiziert.

### Therapie und Verlauf

Um die Tumorausdehnung zu beurteilen, wurden zahlreiche Mappingbiopsien an den sichtbaren Randbereichen durchgeführt. Deren histologische Beurteilung erbrachte eine großflächige Ausdehnung des Tumors vom Kapillitium hochparietal über die Stirn und das obere Mittelgesicht bis retroaurikulär beidseits. Ein daraufhin durchgeführtes Tumorstaging mittels PET(Positronenemissionstomographie)-CT(Computertomographie) zeigte die Ausdehnung des Angiosarkoms am Kapillitium von beidseits okzipital (links > rechts) über temporal nach parietal reichend (Abb. 1f), ergab aber erfreulicherweise keinen Hinweis auf eine Metastasierung. Der Fall wurde in unserem interdisziplinären Hauttumorboard vorgestellt und diskutiert. Es wurde empfohlen, bei nicht resektablem Primärbefund und schwieriger Bestrahlungssituation aufgrund der Beteiligung beider Periorbitalareale eine primäre Chemotherapie mit Paclitaxel 80 mg/m^2^ Q1W einzuleiten. Hierunter kam es rasch zu einer klinischen Besserung mit teilweise wieder möglicher Öffnung des linken Auges (Abb. 1b). Eine Reevaluation mittels PET-CT 8 Wochen nach Therapiebeginn bestätigte die deutliche Abnahme des Glukosemetabolismus im ursprünglichen Tumorareal (Abb. 1g), sodass die Therapie mit Paclitaxel bei partieller Remission fortgesetzt wurde. Fünf Monate nach Therapiebeginn kam es zu einer erneuten Zunahme der periorbitalen Ödeme. Es erfolgte eine Therapieumstellung auf liposomales Doxorubicin (Caelyx 20 mg/m^2^ Q2W). Bei massiver Schwellung der Augenlider mit vollständigem Verschluss des linken Auges nach 4 Zyklen Caelyx (Abb. 1c) wurde aufgrund der raschen Krankheitsprogression die Therapie auf den Multikinaseinhibitor Pazopanib 800 mg/Tag umgestellt. Hierunter war bereits nach wenigen Wochen eine deutliche klinische Besserung bei zunächst guter Verträglichkeit zu verzeichnen. Allerdings entwickelte der Patient 3 Monate nach Therapiebeginn eine ausgedehnte Lungenarterienembolie, aufgrund derer die Therapie mit Pazopanib pausiert werden musste. In der Folge ergab sich rasch ein deutlicher Progress des Angiosarkoms mit erneut fast vollständigem Lidverschluss links, sodass eine weitere Therapie trotz vorliegender Lungenarterienembolie notwendig wurde. Von unserem interdisziplinären Hauttumorboard wurden eine palliative Strahlentherapie sowie die Umstellung der Systemtherapie auf den PD-1-Inhibitor Pembrolizumab empfohlen. Noch vor der geplanten Einleitung dieser Therapie entwickelte der Patient eine Staphylococcus-aureus-Sepsis und verstarb daran, ungefähr 1 Jahr nach der Erstdiagnose des kutanen Angiosarkoms.

## Diskussion

Das kutane Angiosarkom stellt mit einem Anteil von 1–2 % der Weichteilsarkome und 5 % der kutanen Sarkome eine sehr seltene Tumorentität dar [1]. Die Tumoren treten bei ungefähr 50 % der Patienten im Kopf-Hals-Bereich auf [1]. Das idiopathische kutane Angiosarkom betrifft v. a. ältere Menschen mit deutlicher Bevorzugung des männlichen Geschlechts (Verhältnis 3 zu 1) [1]. Die Klinik ist oft unspezifisch und die Prognose aufgrund hoher Lokalrezidivraten und früher hämatogener Metastasierung schlecht [1]. Eine aktuelle Metaanalyse kam zu dem Ergebnis, dass die mittlere Fünfjahresüberlebensrate von Patienten mit Angiosarkom nur 33,5 % beträgt [2]. Weiterhin wurden anhand dieser Analyse ein Lebensalter über 70 Jahre, eine primäre Tumorausdehnung größer 5 cm sowie die Lokalisation im Kopfbereich als Prädiktoren für eine schlechte Prognose identifiziert [2].

Unser Fall führt die Schwierigkeit der Diagnosestellung sowie die Komplexität des Behandlungsverlaufes bei Patienten mit kutanem Angiosarkom vor Augen. Wie bei unserem Fall kommt es beim kutanen Angiosarkom häufig zu einer Verschleppung der Diagnosestellung durch die klinische Ähnlichkeit zu inflammatorischen Dermatosen. So können die Frühstadien mit teigigen Ödemen und Erythemen nicht nur an ein Quincke-Ödem denken lassen, sondern auch an eine Rosazea oder ein Erysipel erinnern. Erschwerend kommt hinzu, dass die Mehrzahl der Patienten asymptomatisch ist und sich erst spät aufgrund von Blutung, Ödemen und/oder Ulzerationen ärztlich vorstellt [3]. Ein weiteres Problem besteht darin, dass es klinisch nur schwer möglich ist, die tatsächliche Tumorausdehnung zu bestimmen. Mappingbiopsien können hier mehr Sicherheit bringen, oftmals wachsen die Tumoren jedoch primär multifokal, was eine operative Sanierung erschwert [1]. Bei eingeschränkter Operabilität stellt die primäre Chemo- und/oder Strahlentherapie die Therapie der Wahl dar. Auch Kinaseinhibitoren, wie in unserem Fall Pazopanib, die die Angiogenese des Tumors hemmen, können vielversprechende Therapieoptionen darstellen. Die Wirksamkeit des Multikinaseinhibitors Pazopanib beim Angiosarkom wurde in mehreren klinischen Studien untersucht. So wurden in der PALETTE-Studie 369 Patienten mit Weichgewebssarkomen nach dem Zufallsprinzip mit 800 mg Pazopanib (*n* = 246) oder Placebo (*n* = 123) behandelt [4]. Das mediane progressionsfreie Überleben in dieser Phase-III-Studie betrug 4,6 Monate für Pazopanib im Vergleich zu 1,6 Monate für Placebo (Hazard Ratio [HR] 0,31; 95 %-KI [Konfidenzintervall] 0,24–0,40; *p* < 0,0001). Das Gesamtüberleben der Patienten betrug 12,5 Monate mit Pazopanib gegenüber 10,7 Monaten unter Placebo (HR 0,86; 95 %-KI 0,67–1,11; *p* = 0,25). Die PALETTE-Studie konnte somit eine Überlegenheit von Pazopanib gegenüber Placebo nachweisen [4], woraufhin die Substanz von der FDA (Food and Drug Administration) für die Indikation Weichteilsarkom zugelassen wurde. Aktuell werden Patienten im Rahmen einer Phase-III-Studie zur Bewertung der Wirksamkeit von Pazopanib zur Behandlung von Patienten mit fortgeschrittenem kutanem Angiosarkom in Japan rekrutiert (JCOG1605, JCOG-PACS).

In unserem vorliegenden Fall war nach Therapieversagen der Standardschemata eine Therapieumstellung auf Pembrolizumab geplant, da eine Therapie mit PD-1-Inhibitoren in Vergleich zu Chemotherapeutika besser verträglich ist. Die Erfahrungen mit Immuncheckpointinhibitoren beim kutanen Angiosarkom und die entsprechende Datenlage sind noch limitiert, was unter anderem daran liegt, dass zwar in zahlreichen Studien die Rolle der Immuntherapie bei Weichteilsarkomen untersucht wird, allerdings seltene Subtypen wie das Angiosarkom oft von einer Teilnahme ausgeschlossen werden. Die Ergebnisse größerer Studien stehen noch aus (NCT02815995), allerdings weisen Fallberichte und Zwischenberichte aus klinischen Studien darauf hin, dass Angiosarkome auf die Immuntherapie ansprechen könnten [5–7]. Die 2016 veröffentlichte multizentrische Phase-II-Studie SARC028 untersuchte die Wirksamkeit und Verträglichkeit des PD-1-Inhibitors Pembrolizumab bei je 40 Patienten mit fortgeschrittenen Weichteil- und Knochensarkomen [8]. Die Ergebnisse zeigten ein Ansprechen auf Pembrolizumab bei 18 % der Patienten mit Weichteilsarkom [8]. Dies deutet auf eine Wirksamkeit von PD-1-Inhibitoren bei der Behandlung von Sarkomen hin. Aktuell werden Sarkompatienten in der SAINT-Studie rekrutiert, in der unter anderem das Zytostatikum Trabectedin und die CTLA-4-Hemmung mit Immuntherapie kombiniert werden (Ipilimumab und Nivolumab, NCT03138161) [9]. Das Angiosarkomprojekt zur genetischen Sequenzierung von Angiosarkomproben hat gezeigt, dass manche kutane Angiosarkome UV-Mutationssignaturen aufweisen, wie sie beim Melanom gefunden wurden [10]. Angesichts der hohen Mutationslast beim Melanom und der relativ hohen Ansprechraten auf die Immuntherapie bietet dies eine mögliche Hypothese, um die Hinweise auf ein Ansprechen von PD-1-Inhibitoren bei kutanen Angiosarkomen zu erklären.

## Fazit für die Praxis

- Der geschilderte Fall verdeutlicht, dass die Planung der Diagnostik und Therapiestrategie bei Patienten mit kutanen Angiosarkomen komplex ist.
- Es empfiehlt sich, diese interdisziplinär abzustimmen sowie die Patienten an ein erfahrenes Sarkomzentrum anzubinden.
